# Kernel approaches for differential expression analysis of mass spectrometry-based metabolomics data

**DOI:** 10.1186/s12859-015-0506-3

**Published:** 2015-03-11

**Authors:** Xiang Zhan, Andrew D Patterson, Debashis Ghosh

**Affiliations:** 1 Department of Statistics, Pennsylvania State University, 325 Thomas Building, University Park, 16802 PA USA; 2 Department of Molecular Toxicology, Pennsylvania State University, 322 Life Sciences Bldg, University Park, 16802 PA USA; 3 Department of Biostatistics and Informatics, Colorado School of Public Health, University of Colorado Anschutz Medical Campus, 13001 East 17th Place, Aurora, 80045 CO USA

**Keywords:** Differential expression analysis, Distance-based kernel, Metabolite, Stratified kernel

## Abstract

**Background:**

Data generated from metabolomics experiments are different from other types of “-omics” data. For example, a common phenomenon in mass spectrometry (MS)-based metabolomics data is that the data matrix frequently contains missing values, which complicates some quantitative analyses. One way to tackle this problem is to treat them as absent. Hence there are two types of information that are available in metabolomics data: presence/absence of a metabolite and a quantitative value of the abundance level of a metabolite if it is present. Combining these two layers of information poses challenges to the application of traditional statistical approaches in differential expression analysis.

**Results:**

In this article, we propose a novel kernel-based score test for the metabolomics differential expression analysis. In order to simultaneously capture both the continuous pattern and discrete pattern in metabolomics data, two new kinds of kernels are designed. One is the distance-based kernel and the other is the stratified kernel. While we initially describe the procedures in the case of single-metabolite analysis, we extend the methods to handle metabolite sets as well.

**Conclusions:**

Evaluation based on both simulated data and real data from a liver cancer metabolomics study indicates that our kernel method has a better performance than some existing alternatives. An implementation of the proposed kernel method in the R statistical computing environment is available at http://works.bepress.com/debashis_ghosh/60/.

**Electronic supplementary material:**

The online version of this article (doi:10.1186/s12859-015-0506-3) contains supplementary material, which is available to authorized users.

## Background

Metabolites are the end products of cellular regulatory and metabolic processes, and their levels can be regarded as the response of biological systems to genetic or environmental changes. Metabolomics experiments have been carried out because of the fundamental regulatory importance of metabolites as components of biochemical pathways, the importance of certain metabolites in human diet and their use as diagnostic markers for a wide range of biological conditions including diseases. Recent advances in experimental techniques like liquid chromatography coupled with mass spectrometry (LC-MS), gas chromatography coupled with mass spectrometry (GC-MS) and capillary electrophoresis coupled with mass spectrometry (CE-MS), allow for the generation of high-throughput metabolomics data. Those platforms support the approach of trying to capture the entire metabolome, rather than only targeted compounds. As a result, the power of metabolomics experiments as a technology platform for gene-function analysis, pharmaceutical research and systems biology, is now beginning to be fully realized [1,2].

Similar to other “-omic” technologies, metabolomics studies require the development of sophisticated and powerful statistical methodologies to evaluate and analyze the data so that it can be turned into further biological insights. While a lot of attention has been paid to the statistical analysis of genomic and proteomic data, less has been given to data from metabolomics experiments. The might be due to the challenges for processing of metabolomics data given the size and complexity of the dataset [3]. For example, mass spectrometry (MS)-based metabolomics data is typically characterized by high dimensionality, small sample size, high correlation structure between metabolites, redundant information (such as adducts and fragments), and especially the sparse data matrix, which is comprised of the samples, the variable ID (m/z, retention time), and peak area. Novel and multiple levels of statistical approaches are required to deal with MS-based metabolomics data. Examples of such statistical analysis can be found in [1] and [4]. In this paper, we focus on differential expression analysis of MS-based metabolomics data. This fundamental approach is to compare the abundance level of a metabolite between an experimental group and a control group, and to use statistics to assess the significance of any differences. This kind of study strongly supports the value of proper identification of putative oncometabolomic markers [5,6].

A big challenge in metabolomics differential expression analysis is that the data matrix frequently contains missing values. The missing values are caused for the following possible reasons. One possible reason is that the peak might have been present in the chromatogram but subsequently missed by peak picking. A second possible reason is that the peak was not initially present in the chromatogram. A third reason is that the peak was present in the chromatogram but the intensity was below the threshold that the equipment can detect [7]. Many statistical methods require a fully defined data matrix. Treatment of missing values is important in metabolomics differential expression analysis. The most straightforward way to handle this missing value problem is to delete a metabolite whenever a missing value is present. However a typical metabolomics dataset has widespread missing values, so deleting metabolites with missing values will result in a much smaller dataset. Another way to deal with missing value problem is through the use of imputation methods. However, as mentioned above, the pattern of the missing values is very complicated. Simple imputation routines often need the assumption of missing at random [8], which is probably not appropriate in metabolomics experiments. Another argument against imputation is that one may think that, by imputing the missing values, it only sidesteps the problem instead of incorporating this missingness as a fundamental feature of metabolomics data [9].

An alternative is to code the missing values as zeroes, denoting absence. Based on that, some methodologies are proposed. One example is the presence/absence analysis for proteomics data proposed in [10]. In their analysis, the data matrix is digitized into binary measurements depending on whether a peptide is present or not. In this paper, a kernel-based score test is introduced to perform the differential analysis in metabolomics experiments. We also code missing values as zeroes; however, we keep the non-zero values instead of coding them to ones. Note that two types of information are contained in a typical metabolomics dataset. One is the presence and absence information of a metabolite and the other is a quantitative abundance level of a metabolite if it is present. This special structure poses challenges to many existing statistical methodologies, which often fail to utilize both the continuous and discrete nature of metabolomics data simultaneously. To address this issue, two new kinds of kernels are designed to take the special pattern in metabolomics data into account. Beyond the point of incorporating missing values in metabolomics experiments as an important aspect of the data, another contribution of this paper is that we group metabolites into metabolite-sets and perform the kernel-based score test on those metabolite-sets. This allows the unit of analysis to be both a single metabolite and a metabolite-set with multiple metabolites, which is more flexible than traditional analysis. Correlation coefficients are used for grouping the metabolites. Our methods are evaluated via simulation studies and by application to real data from a liver cancer metabolomics study.

## Methods

### Kernels

Most statistical strategies used in differential expression analysis are based on linear methods like OLS (ordinary least squares regression) and ANOVA (analysis of variance). Linear methods are very popular in many statistical problems, mostly because the simple mathematical form allows simple algorithms and detailed study of their properties. Real-world data problems, on the other hand, often require nonlinear methods. Taking metabolomics differential expression analysis as an example, linear methods are not able to simultaneously capture both the continuous and the discrete nature of metabolomics data. Kernels provide a way to extend those linear methods to nonlinear ones in a computationally tractable manner. Examples of a gain in terms of a certain criterion by extension from linearity to non-linearity can be found in [11,12]. For those readers who are interested in the topic of kernel methods, more details can be found in [13] and [14]. Broadly speaking, instead of assuming a linear functional relationship, kernel-based methods assume a more general relationship based on a Hilbert space of functions spanned by a certain kernel function.

Let  be the input space (usually a compact subspace of *R* ^*p*^). In this paper, we call a bivariate symmetric function *k*(*x*,*y*) on $\mathcal {X} \times \mathcal {X}$ is a kernel or a kernel function if

$$\int_{\mathcal{X}} \int_{\mathcal{X}} k(x,y)g(x)g(y)dxdy \geq 0, $$

for all squared integrable function *g*(·) on , i.e., $g(\cdot) \in L^{2}(\mathcal {X})$. Equivalently, for any finite collection of points $x_{1},\ldots,x_{n} \in \mathcal {X}, n \ge 2$, the Gram (kernel) matrix *K* with *K*_*ij*_=*k*(*x*_*i*_,*x*_*j*_) is positive definite.

An important concept relating to kernel methods is that of reproducing kernel Hilbert space (RKHS). The Moore-Aronszajn theorem states that, for each kernel *k* on a set , there is a unique Hilbert space of functions on  for which *k* is a reproducing kernel [15]. Mercer’s theorem characterizes the relationship between a RKHS and its reproducing kernel. Details can be found in [16]. Kernel methods have been widely used in genome-wide association studies (GWAS). Examples include [11,17-19]. It works in the following way. Let *Y* be the phenotype and *X* be the genotype variables. Let *i*,*j* be two subjects in the study. Because *k*(·,·) is positive definite, *k*(*X*_*i*_,*X*_*j*_) can be used as a measure of similarity between individual pair (*i*,*j*) in terms of genotype variables *X*. Then, this kernel-based similarity measure can be incorporated to test to what extent variation in the level of similarity exhibited by pairs of individual can explain the similarity in *Y*. In the next subsection, we propose an approach to apply the kernel to our metabolomics differential expression analysis in a similar way as done in the genomics studies.

One of the most commonly used kernels is the Gaussian kernel *k*(*x*,*y*)= exp{−*ρ* ^−1^||*x*−*y*||^2^}, where *ρ* is a positive parameter and ||·|| is the *L* ^2^ norm. The Gaussian kernel generates the function space spanned by radial basis functions (RBF) and *ρ* is called bandwidth or shape parameter in literature [20]. The RKHS generated by a Gaussian kernel includes a very broad class of functions. Another widely used kernel is the *d*th polynomial kernel given by *k*(*x*,*y*)=(*x* ^*T*^ *y*+*ρ*)^*d*^, where *ρ*≥0 and *d* is a positive integer. It is called the homogeneous polynomial kernel if *ρ*=0. Other examples include spline kernels, ANOVA kernels, tree kernels and graph kernels [21].

### A kernel-based score test for differential expression analysis

Traditional metabolite differential analysis often involves analyzing each metabolite individually in a parametric model, which generates a p-value for each metabolite [1]. Then significance of a metabolite is claimed if its p-value is smaller than some certain threshold, which is often corrected for multiple testing. This kind of individual analysis can be useful. However it is widely observed in genomic study that individual analysis can be limited [17,18]. One appealing feature of the kernel-based score test we propose in this paper is that, it allows the unit of analysis to be shifted from individual metabolite to groupings of metabolites. Hence metabolic pathway analysis is also allowed in our framework.

For a fixed metabolite-set, we observe the data {*x*_*i*_,*y*_*i*_},*i*=1,…,*n*, where *n* is number of observations. *x*_*i*_ is the abundance level of the metabolites for sample *i*, and it can be either a vector (if the metabolite-set contains more than one metabolite) or a scaler (if the metabolite-set consists of only one metabolite). The dimension of *x*_*i*_ equals the size of the metabolite-set. *y*_*i*_ is the case-control label for observation *i*. For ease of exposition, we assume that *y*_*i*_ is binary so that only two groups are being considered. Moreover, *y*=1 indicates the case group and *y*=0 indicates the control group. The following logistic model is assumed in this paper:

(1) $$ logit \left[Pr(y_{i}=1)\right]= \beta_{0}+ f(x_{i}),  $$

where *f*(·) is a centered smooth function in a RKHS spanned by kernel *k*. Unlike traditional models, Model (1) is semi-parametric in the sense that it does not put any parametric assumption on *f*(·) except that it is assumed to lie in a certain functional space. Hence, kernel-based models are more flexible and more robust to issues like model misspecification. Model (1) looks at the problem of finding differentially expressed metabolite-sets from a different point of view. If *f*(·)=0, then metabolite expression value *x*_*i*_ is not related to the group labels *y*_*i*_. Hence, a differentially expressed metabolite-set will lead to a rejection of the hypothesis *H*_0_:*f*(·)=0.

A similar test has been discussed in [11]. Following the discussion there, a score test for differential analysis with metabolites can be proposed as follows. Let K be the *n*×*n* Gram matrix with *K*_*jk*_=*k*_*ρ*_(*x*_*j*_,*x*_*k*_). Here *k*_*ρ*_(·,·) is the reproducing kernel of the RKHS which contains *f*(·), and *ρ* is an unknown kernel parameter. Let **y** be the *n*×1 vector of 0/1 group labels. The test statistic for *H*_0_:*f*(·)=0 in model (1) is

(2) $$ S(\rho)=\frac{Q(\rho)-\mu_{Q}}{\sigma_{Q}},  $$

where $Q(\rho)=(\boldsymbol {y-\hat {\mu }_{0}})^{T}K(\boldsymbol {y-\hat {\mu }_{0}})$, $\hat {\mu }_{0} = logit^{-1} \hat {\beta }_{0}$ and $\hat {\beta }_{0}$ is the estimate of *β*_0_ under the null *H*_0_:*f*(·)=0 in model (1). *μ*_*Q*_ and ${\sigma ^{2}_{Q}}$ are the mean and variance of *Q*(*ρ*) respectively (more details about *μ*_*Q*_ and ${\sigma ^{2}_{Q}}$ can be found in [11]). Note that *f*(·) is in the RKHS spanned be *k*_*ρ*_(·,·). That is, *ρ* does not enter Model (1) under the null *H*_0_:*f*(·)=0. Hence, the test statistic *S*(*ρ*) is inestimable under *H*_0_ and the significance level cannot be established. Davies, 1977 [22] and Davies, 1987 [23] considered this non-standard testing problem and proposed a test based on the process {*S*(*ρ*),*ρ*∈[*L*,*U*]}. This test has rejection region of the form {sup*L*≤*ρ*≤*U* *S*(*ρ*)>*c*}. Using this test, an upper-bound for the p-value of testing *H*_0_:*f*(·)=0 in (1) is given by

(3) $$ \Phi(-M)+V \exp\left(\frac{1}{2}M^{2} \right)/\sqrt{8\pi},  $$

where *Φ*(·) is the cumulative distribution function of the standard normal density, *M* is the maximum of *S*(*ρ*) over the range of *ρ* and *V*=|*S*(*ρ*_1_)−*S*(*L*)|+|*S*(*ρ*_2_)−*S*(*ρ*_1_)|+⋯+|*S*(*U*)−*S*(*ρ*_*m*_)| is the total variation of *S*(*ρ*) over the interval [*L*,*U*], where *ρ*_1_,…,*ρ*_*m*_ are *m* grid points in the interval [*L*,*U*]. For the kernels used in this paper, the parameter *ρ* takes values in (0,*∞*). Based on the discussion in [11], it is not necessary to consider all *ρ* values up to *∞*. For computational purposes, we take [*L*,*U*] to be [10^−3^,10^3^] and *m*≡200 evenly spaced grid points are used.

The test statistic $Q(\rho)=(\boldsymbol {y-\hat {\mu }_{0}})^{T}K(\boldsymbol {y-\hat {\mu }_{0}})$ can be rewritten as

$$ Q(\rho)=\sum_{i=1}^{n} \sum_{j=1}^{n} k\left(x_{i},x_{j}\right)\left(y_{i}-\hat{\mu}_{0}\right)\left(y_{j}-\hat{\mu}_{0}\right), $$

which is the componentwise product of the matrices *K* and $\boldsymbol {(y-\hat {\mu }_{0})(y-\hat {\mu }_{0})^{T}}$. Note that $\boldsymbol {\hat {\mu }}_{0}$ is the expectation of ***y*** under the null hypothesis, so the matrix $\boldsymbol {(y-\hat {\mu }_{0})(y-\hat {\mu }_{0})^{T}}$ is the covariance matrix of the outcomes under the null. If we replace the kernel matrix *K* with the covariance matrix of *X*, then this score test is essentially the one considered in [24]. Because kernel *k*(·,·) is positive definite, the kernel matrix K with *K*_*ij*_=*k*(*x*_*i*_,*x*_*j*_) can be viewed as a generalization of the covariance matrix of metabolite-expression patterns between the samples. Therefore, the kernel-based score test is a generalization of the association test in [24] and it enjoys the locally optimal power property in [25]. The test statistic *Q*(*ρ*) has a high value if the covariance structure of metabolite-expression resembles that of the outcome. The remaining task is to design a kernel that can serve as a proper covariance matrix of *x*. Two such kernels have been designed to take the special structure of metabolomics data into account.

### Kernels for metabolomics data

In this section, we always assume that the metabolite-set is fixed and contains *p* metabolites. An measurement of the metabolite-set on one subject contains *p* individual metabolite measurements. Each metabolite measurement can be positive (indicating the metabolite abundance level) or zero (indicating absence of that metabolite). Let $x, y \in \mathcal {X}^{p}$ be two arbitrary metabolite-set measurements, where $\mathcal {X}\equiv R^{+}\cup \{0\}$. Note that we use notation *y* as metabolite-set measurements in this section, while it was used as group indicator in the previous section. In what follows, the *y* in function *k*(*x*,*y*) or *d*(*x*,*y*) always denotes metabolite measurements unless specified differently.

#### Distance-based kernel

One commonly used kernel is the Gaussian kernel:

$$k(x,y) = \exp\left\{-\frac{\sum_{i=1}^{p}(x_{i}-y_{i})^{2}}{\rho}\right \}; \: x,y \in R^{p}, $$

where *ρ*>0 and *x*_*i*_ is the *i*th dimension of *x*. Inspired by the Gaussian kernel, we define a distance between two measurements *x* and *y* as:

(4) $$ d(x,y)=\sqrt{\sum_{i=1}^{p} I_{\left[\delta_{x_{i}} \neq \delta_{y_{i}}\right]}+\sum_{i=1}^{p}(x_{i}-y_{i})^{2}},  $$

where $x_{i} \in \mathcal {X}, i=1,\ldots,p$ is measurement of the *i*th metabolite in the metabolite-set, and $\delta _{x_{i}}= I_{\left [x_{i} \neq 0\right ]}$ is the indicator of the presence of the *i*th metabolite. The first term in Eq. (4) captures the discrete nature of metabolomics data, and the second term captures the continuous nature of metabolomics data. Compared to the Euclidean distance on *R* ^*p*^, we modify the Euclidean distance by adding the first term to define a distance on the set $\mathcal {X}^{p}$. Think about the new distance in a simple example. Suppose the measurements are now scalars (there is only one metabolite in each metabolite-set). Take three measurements *x*_1_=0, *x*_2_=1 and *x*_3_=2 as an example. $d(x_{1},x_{2})=\sqrt {2}$ while *d*(*x*_2_,*x*_3_)=1. The first term in the distance defined in Eq. (4) can be taken as a penalty term for being absent. Based on this distance, a kernel is constructed as follows,

(5) $$ k_{d}(x,y)= \exp \left\{-\frac{d^{2}(x,y)}{\rho} \right\}.  $$

The proof that (4) is a well-defined distance metric and (5) is well-defined kernel can be found in the Supplementary materials (Section 1 in Additional file 1). Readers who are less interested in the mathematical details can skip the proof. There is little harm in understanding the kernel-based score test proposed in this paper by simply treating the distance-based kernel as a variant of the Gaussian kernel. Due to the way we define the distance Eq. (4), the distance-based kernel *k*_*d*_(*x*,*y*) can take both continuous nature and discrete nature of metabolomics data into account. Some kinds of data processing, like normalization, is recommended when using this kind of kernel. For example, if those nonzero numerical values are relatively large, then the continuous pattern will dominate the other one in Eq. (4). Conversely, when the continuous values are relatively small, then the second term will have less weight compared to the first one.

#### Stratified kernel

An alternative way to define a kernel on $\mathcal {X}^{p}$ is using stratification. We first partition $\mathcal {X}^{p}$ into smaller sets. On each of those partitions, it is easier to define a kernel function. Then we use the stratified representation proposed in [26] to build a kernel on $\mathcal {X}^{p}$. Let *Ω* be a general complicated set, on which it is difficult to define an appropriate kernel function. Suppose $\Omega = \bigcup _{n=1}^{m} \Omega _{n}$, where *Ω*_1_,…,*Ω*_*m*_ are disjoint partitions of *Ω*, and *m* is the number of partitions. Let $\{k^{n} (\cdot,\cdot)\}_{n=1}^{m}$ be a family of kernels on *Ω*_*n*_×*Ω*_*n*_,*n*=1,…,*m*. For *ω*_*i*_, *ω*_*j*_∈*Ω*, define

$$k_{s}(\omega_{i},\omega_{j})= \left\{ \begin{array}{ll} k^{n} (\omega_{i},\omega_{j}) &\quad \text{if both \(\omega_{i},\omega_{j} \in \Omega_{n}\)}, \\0 & \quad \text{otherwise.} \end{array} \right. $$

Park et al., 2012 [26] showed that *k*_*s*_(·,·) is strictly positive-definite as long as each kernel *k* ^*n*^(·,·),*n*=1,…,*m* is a bounded strictly positive-definite kernel. We extend this result to our metabolite differential expression analysis by proposing the partitions as follows. Let $\mathcal {X}_{1}^{p}\equiv \{0\}^{p}$ denote the stratum that no metabolite is present; $\mathcal {X}_{2}^{p}\equiv R^{+}\times \{0\}^{p-1}$ denote the stratum that only the first metabolite in the set is present while all others are absent; $\mathcal {X}_{3}^{p}\equiv \{0\} \times R^{+}\times \{0\}^{p-2}$ denote the stratum that only the second metabolite in the set is present while all others are absent; and $\mathcal {X}_{2^{p}}^{p} \equiv \{ R^{+} \}^{p} $ denote the one that all metabolites in the set are present. Notice that $\mathcal {X}_{i}^{p}$’s are disjoint, and

$$\mathcal{X}^{p} = \bigcup_{i=1}^{2^{p}} \mathcal{X}_{i}^{p}. $$

That is, $\mathcal {X}^{p}$ is partitioned into 2^*p*^ pieces depending on whether each metabolite in the metabolite-set is present or not. Under this stratified representation, if $x,y \in \mathcal {X}^{p}$ come from the same stratum, then they have exactly the same subset of metabolites being present (or absent). Hence, we only need to consider the continuous aspect information if two measurements are within the same stratum. If they are from different stratums, we just assign a 0 value for the kernel function. For $x, y \in \mathcal {X}^{p}$, the stratified kernel *k*_*s*_(*x*,*y*) is defined as

(6) $$ k_{s}(x,y)= \left\{ \begin{array}{ll} k^{i} (x,y) & \quad \text{if both}\,\, x, y \in \mathcal{X}_{i}^{p} \\ 0 & \quad \text{otherwise} \end{array} \right.,  $$

where *i*=1,…,2^*p*^, and *k* ^*i*^(*x*,*y*) is a kernel defined on $\mathcal {X}_{i}^{p}$. In this paper, the Gaussian kernel is used for *k* ^*i*^(*x*,*y*). Since the Gaussian kernel is bounded and strictly positive-definite, *k*_*s*_(*x*,*y*) defined in Eq. (6) is then strictly positive-definite based on result in [26].

Unlike the distance-based kernel, the stratified kernel utilizes both the continuous nature and discrete nature of metabolomics data in stratification. We first use the discrete aspect of information to define each stratum. Then, within each stratum, the presence/absence information of each measurement are the same. After this presence/absence information being taken account for, we use a relatively simpler kernel *k* ^*i*^(*x*,*y*) to capture the continuous pattern in the data. An advantage of this stratified kernel is that it is a little computationally cheaper than the distance-based kernel, because the entries of the stratified kernel matrix are simpler to calculate. However, the disadvantage of this kind of kernel is also straightforward. When *p* is large, the number of samples in each stratum is small, and most entries of the kernel matrix are zeroes. This may lead to a less informative kernel matrix which fails to capture the correlation structure between metabolites.

### Forming metabolites set and multiple testing correction

The mode of our metabolite differential analysis proceeds in two steps. First, metabolites are assigned to metabolite-sets based on some certain criteria. Second, a kernel-based score test is performed on each set. A nice feature of our method is that metabolite-set analysis is allowed in our framework. The advantages of genetic set/pathway analysis have been widely explored in the kernel literature [11,17,18]. A similar phenomena is also observed for metabolomics data [27-29]. In our metabolomics differential expression analysis, there are two major appealing advantages of allowing set-based analysis. First, tests based on metabolite-sets tend to have greater power. This is because, one can borrow information from different but correlated metabolites and take advantage of the correlation between individuals, by pooling individual metabolites into sets. Second, the number of hypotheses being tested is reduced and thus relaxes the stringent condition needed for correction of multiple testing. This could be very helpful in those metabolomic experiments which detected a large number of peaks, features or compounds.

In the data experiments, the grouping criterion we used is based on correlation coefficients. We first picked a correlation threshold value *c*. Each metabolite was treated as a node and we added an edge between two nodes if the absolute correlation value between these two nodes was greater than the threshold *c*. In the end, those nodes which were connected were assigned in the same set. This group scheme is based on the following reasoning. We think that, for each metabolite in a metabolic pathway, there exists at least another metabolite which is strongly correlated with it. We also tried setting threshold value on pairwise correlations. That was, each pair of metabolites in a set had an absolute correlation coefficient greater than the threshold value. However, we found that pairwise correlation was too stringent and resulted in metabolite-sets that were too small. This seems plausible, as it is unlikely to be the case that for metabolite pathways in which every pair of metabolites are strongly correlated. Other grouping criterion are also proposed recently by Suvitaival et al. in [27-29]. The motivation of both our grouping method and the methods proposed by Suvitaival et al. are the same. We both observe that multiple peaks are highly correlated, and both want to increase the statistical strength of differential analysis by grouping. They differ in the grouping mechanisms. In our method, the grouping is done based on simple network model, while in their’s, grouping is based on more complicated hierarchical Bayesian models. Other schemes like grouping based on some meaningful biochemical pathway knowledge are also possible. In principle, our kernel approach is statistically valid irrespective of the grouping scheme. The choice of determining optimal groupings is an open one and beyond the scope of the current investigation.

Note that we applied a kernel-based score test to each metabolite-set. Assessing differential expression in this setting leads to a multiple comparisons involving results from hundreds of univariate kernel score tests. We need to apply some sort of multiple testing correction to control a certain overall error measure such as false discovery rate (FDR) [30]. Following [31], the FDR associated with a rejection p-value cutoff *c* (i.e., reject an individual test with a p-value smaller than *c*) is the expected of false positives F(c) out of the total number of positives S(c):

(7) $$ FDR(c)= E\left[\frac{F(c)}{S(c)}\right] \approx \frac{E\left[F(c)\right]}{E\left[S(c)\right]}  $$

*E*[*S*(*c*)] is simply estimated by the observed number of positives. *E*[*F*(*c*)] is estimated by $p\hat {\pi }_{0}(\lambda) c$, where *M* is the total number of hypotheses, $\hat {\pi }_{0}(\lambda)$ is the estimated proportion of true null given by

(8) $$ \hat{\pi}_{0}(\lambda)= \frac{\sum_{i=1}^{M} I_{\left[p_{i}>\lambda\right]}}{M(1-\lambda)},  $$

where *λ* is a tuning parameter between 0 and 1. According to [31], Eq. (8) holds when the true null p-values are almost uniformly distributed. Therefore, given a rejection region of [0,*c*] based on p-values of individual tests, the FDR was estimated as

(9) $$ \hat{FDR}(c) = \frac{M\hat{\pi}_{0}(\lambda)c} {\sum_{i=1}^{M} I_{\left[p_{i} \leq c\right]}}.  $$

Eq. (9) will be used when we need to estimate FDR. In the simulation studies, we know whether an individual hypothesis is a true null hypothesis or a true alternative hypothesis. Hence, the true FDR can be calculated and we do not need Eq. (9). As pointed out in [31], an assumption that Eq. (9) holds is that the p-values of the true null hypotheses are almost uniformly distributed. This assumption will be checked before applying Eq. (9) in the real data experiment analysis. Moreover, Eq. (9) also relies on the tuning parameter *λ*. We tried different *λ* values in our data experiments. Last, [10] propose a hybrid procedure to estimate *F* *D* *R*(*c*) in Eq. (7) using a binary weight (instead of the uniform weight as in Eq.(8)). This approach is well-suited to the application described in [10]. However, unlike Eq. (9) proposed in [31], the theoretical properties of this hybrid method are not well-understood. Therefore, we do not attempt comparisons with the hybrid method to estimate FDR in this paper.

## Results and discussion

### Simulated data

Wang et al., 2012 [10] and Karpievitch et al., 2009 [32] propose different methods for proteomics differential expression analysis. Since MS-based proteomics datasets and MS-based metabolomics datasets share some common characteristics including the widespread missing values. We are also interested in applying those two methods to our metabolomics differential expression analysis. We would like first to give a brief description of those methods before applying them in our simulation studies.

In [32], a likelihood-based method is proposed. In particular, let *y*_*ijkl*_ be the intensity for protein *i*, peptide *j* in group *k* and sample *l*. The following additive linear model is assumed.

$$y_{ijkl} = {Prot}_{i}+{Pep}_{ij}+{Grp}_{ik}+{error}_{ijkl}, $$

where *P* *r* *o* *t*_*i*_ represents the overall intensity for protein *i*, *P* *e* *p*_*ij*_ represents the effect of peptide *j* in protein *i*, *G* *r* *p*_*ik*_ represents the effect of group *k* in protein *i*, and ${error}_{\textit {ijkl}} \sim N(0,\sigma ^{2}_{\textit {ij}}$). The sum-to-zero constrain is applied to both peptide effects and group effects, that is, $\sum _{j} {Pep}_{\textit {ij}}=0$, and $\sum _{k} {Grp}_{\textit {ik}}=0$. The relevant null hypothesis for testing differential expression in protein *i* is *H*_0*i*_:*G* *r* *p*_*i*1_=⋯=*G* *r* *p*_*iK*_=0 (assuming there are K groups in total). Based on this linear model, $y_{\textit {ijkl}} \sim N(\mu _{\textit {ijk}}, \sigma ^{2}_{\textit {ij}})$, where *μ*_*ijk*_=*P* *r* *o* *t*_*i*_+*P* *e* *p*_*ij*_+*G* *r* *p*_*ik*_. Then the author propose a likelihood function considering both the likelihood contribution of unobserved *y*_*ijkl*_ measurements and that of observed *y*_*ijkl*_ measurements (see [32] for more details). Finally, a likelihood ratio test statistic is used for testing differential expressions.

As an alternative to the intensity-based analysis in [10,32] propose a presence/absence analysis, in which peak intensities are digitized into binary measurements depending on whether a peak was observed or not. A logistic regression model is used for this presence/absence analysis. Specifically, let *y*_*ijkl*_ be the indicator for whether a peak was observed for protein *i*, peptide *j* in group *k* and sample *l* (note that the notation *y*_*ijkl*_ is different from the one used in [32]). Then *y*_*ijkl*_∼*B* *e* *r* *n* *o* *u* *l* *l* *i*(*p*_*ijk*_), and the logistic regression model is:

$$ logit(p_{ijk})= {Prot}_{i}+{Pep}_{ij}+{Grp}_{ik}, $$

where the notations for the parameters *P* *r* *o* *t*_*i*_, *P* *e* *p*_*ij*_, *G* *r* *p*_*ik*_ are the same as those in [32], and the same sum-to-zero constrains are also applied in this logistic model. The null hypothesis for testing differential expression in protein *i* is *H*_0*i*_:*G* *r* *p*_*i*1_=*G* *r* *p*_*i*2_=0 (assuming there are only 2 groups in this presence/absence analysis). The proposed test statistics is

$$ T_{i}=\left|\sum_{j=1}^{m_{i}}\omega_{ij}(y_{ij1\cdot}-y_{ij2\cdot})\right|, $$

where *m*_*i*_ is the number of peptides in protein *i*, $\omega _{\textit {ij}}=y_{ij\cdot \cdot }/\sum _{j} y_{ij\cdot \cdot }$, and a · indicates a summation over that index. *T*_*i*_ is a weighted average of observed presence difference *T*_*ij*_ (where *T*_*ij*_=*y*_*ij*1·_−*y*_*ij*2·_) on each sibling peptide *j*. Finally, A bootstrap-based test on *T*_*i*_ is proposed for *H*_0*i*_:*G* *r* *p*_*i*1_=*G* *r* *p*_*i*2_=0 (see [10] for more details).

We conducted simulation studies to evaluate and to compare the performance of various tests including two kernel-based score tests and the two tests used in proteomics differential expression analysis. Simulation data were generated in a similar way as in [10]. Specifically, we generated our simulated data with 1000 metabolite-sets and 20 samples. The number of metabolites within each metabolite-set was uniformly distributed between 1 and 15. The group information had already been incorporated in the data, we omitted the grouping stage of our kernel method to make sure that all methods were working on the same data structure. More details about this simulation setup could be found in [10]. This data was summarized in a matrix with each row representing a metabolite and each column representing a sample. Each cell in the data matrix was a measurement of metabolite abundance level. Different rows formed a metabolite-set and our analysis was performed at the level of metabolite-set.

In a typical metabolomics experiment, about 20% to 40% total collection of measurements are missing, i.e., 20% to 40% cells in the data matrix are zeroes. We first generated the complete data matrix. A certain proportion of the smallest values in this complete data matrix were set to be 0, indicating absence of a metabolite. This is reasonable because one major reason for missing values in MS based metabolomics study is that the abundance level is lower than the detection threshold of the device. In our simulation, the proportion of zeroes was 20% or 40%. The complete data were generated from the following linear fixed effects model:

$$X_{ijkl} = S_{i}+ M_{ij}+ G_{ik}+ {Err}_{ijkl}, $$

where *i*=1,…,1000 were metabolite-sets, *j*=1,…,*n*_*i*_ were metabolites within the *i*th metabolite-set, *k*=1,2 were group labels and *l*=1,…10 were samples or replicates within each group. In this linear model, *S*_*i*_∼*u* *n* *i* *f* *o* *r* *m*(10,14) was the effect of metabolite-set *i*; *M*_*ij*_∼*u* *n* *i* *f* *o* *r* *m*(−2,2) was the effect of metabolite *j* in set *i*. *G*_*ik*_ was the treatment (group) effect in metabolite-set *i*. Here, we were more interested in the treatment effect on the whole metabolite-set instead of an individual metabolite. That was why we omitted the subindex *j* in *G*_*ik*_; *E* *r* *r*_*ijkl*_ was normally distributed error term with mean 0 and different variances for different metabolite-sets. For model identifiability, we assumed that *G*_*i*2_=0. The null hypothesis *H*_0_:*G*_*i*1_=0 was of our interest in this differential expression analysis. We considered both a high and low level magnitude of group effect. For the high-level magnitude group effect *G*_*i*1_=−3 or −4, and for the low-level magnitude group effect *G*_*i*1_=−1 or −2. Finally, the proportion of the true null hypotheses were 75% and 50%. Here we called metabolite *i* a true null hypothesis if it was not differentially expressed, i.e., *H*_0*i*_:*G*_*i*1_=*G*_*i*2_=0. To sum up, we had in total 8 different simulation scenarios: two different levels of group effect (high and low), two different levels of missing data (20% and 40%) and two different proportions of differentially expressed metabolites (25% and 50%). Under each scenario, one simulated dataset of 1000 metabolite-sets expressions from 20 samples in two groups was generated. The size of each metabolite-set varied from 1 to 15.

We applied both the distance-based kernel score test and stratified kernel score test. Because the range of *X*_*ijkl*_ was small, we did not use any normalization when applying the distance-based kernel score test based on Eq. (4). Other existing methods such as the qualitative presence/absence method [10] and the quantitative intensity-based method [32] were also used for purposes of comparison. As a benchmark, two-sample t-test, which is commonly used in differential expression analysis, was also performed in this simulation study. When the size of metabolite-set was greater than 1, an extension of the t-test, the Hotelling *T* ^2^ test was applied. In our simulations, the Hotelling *T* ^2^ could not be computed because the estimated variance matrix was not invertible due to the widespread zero values in metabolomics data. To fix such an issue, we averaged the abundance level over all metabolites in the set and applied a t-test to the one-dimensional average score. Metabolomics data are often highly skewed, which violates the normality assumption in t test. Hence, we also applied the Wilcoxon signed-rank test [33]. One similar issue is that the ranking process required in Wilcoxon method does not work when the metabolite-set contains more than one metabolite. In such a case, as what we did in t test, the averaged score of abundance level was calculated and the Wilcoxon signed-rank test was applied to this one-dimension score. For simplicity, we refer to these tests as Kernd, Kerns, Qual, Quant, T and Wilcox, respectively, in what follows.

**Figure 1:**
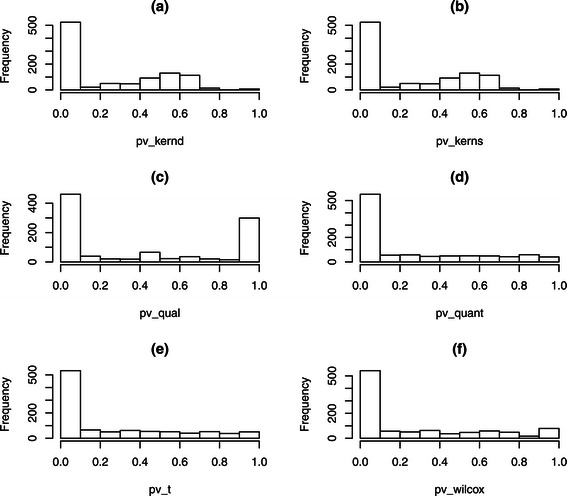
**Histogram of p-values.** Histogram of p-values on the simulated data with low group effect, 20% missing data and 50% differentially expressed metabolites. The titles **(a)**, **(b)**, **(c)**, **(d)**, **(e)**, **(f)** correspond to methods Kernd, Kerns, Qual, Quant, T and Wilcox respectively.

**Figure 2:**
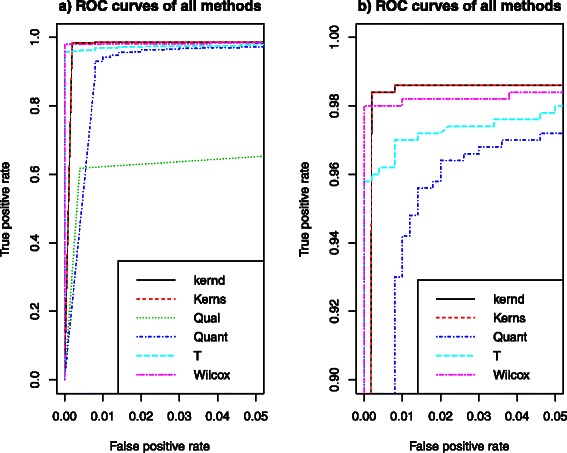
**ROC curve.** ROC curves of different methods on the simulated data with low group effect, 20% missing data and 50% differentially expressed metabolites. The range of y-axis on the left panel **a)** is [0,1], and the range of y-axis on the right panel **b)** is [0.9,1].

Figure 1 presents histograms of p-value of different methods on the simulation scenario with low group effect, 20% missing data and 50% differentially expressed metabolites. In this simulation, a total of 1000 metabolite-sets were generated, and the first 500 metabolite-sets were truly differentially expressed. Except for the Qual method, the p-value histograms of all other five methods showed an expected distribution in the sense that about 500 hypotheses were rejected and most of them were true positive (the corresponding number in Qual method was about 400 instead). Figure 2 are the ROC curves of different methods on the same simulation study as that in Figure 1. We were interested in the performance of all tests in a low false positive rate. Hence we considered FDR ≤0.05. Based on Figure 2a) (left panel), kernel methods, Quant, T and Wilcox methods had better performance than the Qual method, which supported the result found in Figure 1. We focused on the area with true positive rate form 0.9 to 1 to get the Figure 2b) (right panel). Because Qual method could not achieve a true positive rate greater than 0.8 based on Figure 2a), Qual did not appear in Figure 2b). Based on Figure 2b), we can see that kernel methods had best performance in terms of ROC curve, followed by the Wilcoxon signed-rank test. A similar pattern was observed for other simulation scenarios. One reason for such a good performance of Wilcoxon test is that there were almost no ties in our simulation setting. Recall that the number of metabolites in each set was uniformly distributed in {1,2,…,15}, and most of the 1000 metabolite contained more than one metabolite. We performed the Wilcoxon test on the average score of multiple metabolites. However, by taking average value for metabolites, it broke down many tied zero values. Hence, in the average scores, there were much fewer ties. That explained the fairly good performance of Wilcoxon test presented in Figure 2.

Table 1 shows the number of identified significantly differentially expressed metabolite-sets at a true FDR level of 0.05 of different methods under various simulation settings. The notation 25% diff and 50% diff indicate that there are 250 and 500 truly differentially expressed metabolite-sets respectively. 20% and 40% of missingness (proportion of zeroes in the data matrix) are considered in Table 1. Based on Table 1, the Qual method has too many false positives. Irrespective of the cutoff point *c*, the qualitative method cannot achieve a true FDR smaller than 0.05. The reason for its performance of may be that it worked on a less informative binary matrix while the other five methods worked on a semicontinuous matrix. The Quant method was not effective when the proportion of missing data was large and the number of truly differential expressed gene was small. Our kernel score tests, the T-test and the Wilcoxon test worked quite well in all simulation scenarios. The kernel tests seemed to have a slight advantage over the T-test and the Wilcoxon test across different scenarios especially when the group effect was low. The major reason for the good performance of our kernel methods it that the kernels are well designed to take both aspects of information in metabolomics data into account. Another advantage of kernel methods is its flexibility. Note that the kernel methods do not assume any parametric assumptions, while three other alternative methods (Qual, Quant, T) rely on the linearity assumption. Even though we generated our data under a linear model, yet our kernel methods still had a slight advantage over those parametric methods. Besides the flexibility in model assumption, our kernel methods are also more flexible in application. The Hotelling *T* ^2^ test breaks down because of widespread zeroes leading to singularity of the sample covariance matrix, and the Wilcoxon signed-rank test breaks down when the dimension of metabolite-set is greater than 1. However, our kernel method are able to handle both cases without any adjustments.

**Table 1:** **Number of identified significantly differentially expressed metabolites at a true FDR level of 0.05**

**Low group effect**	**25% diff**		**50% diff**	
**Method/miss(%)**	**20%**	**40%**	**20%**	**40%**
Kernd	259	247	517	500
Kerns	259	247	517	500
Qual	*	*	*	*
Quant	244	*	511	487
T	255	241	515	496
Wilcox	254	245	517	496
**High group effect**	**25% diff**		**50% diff**	
**Method/miss(%)**	**20%**	**40%**	**20%**	**40%**
Kernd	260	248	523	511
Kerns	260	248	523	511
Qual	*	*	*	*
Quant	263	*	525	525
T	260	249	523	510
Wilcox	259	247	523	509

The symbol * in the table means that the method can not obtain a true FDR of 0.05 no matter what rejection region [0,c] is used.

### Real data

The data analyzed in this section come from a metabolomics study on the effect of hepatocellular carcinoma (HCC) on liver metabolism and circulating endogenous metabolites. This dataset is publicly available, and that details of the study including the statements of ethical approval and informed consent are provided in the original paper [5]. Ultraperformance liquid chromatography coupled with electrospray ionization quadrupole time-of-flight mass spectrometry was used to profile the samples. The raw chromatographic and spectral data were aligned, deconvoluted and normalized by Pareto scaling. We do not perform any other normalization in the distance-based kernel score test. A total of 1388 features have been generated on 55 subjects. Each spectral feature was represented by a unique m/z, retention time, and peak area. For clarity and simplicity, we define a feature a unique m/z and retention time. Among those 55 subjects, 20 are HCC patients, 35 are non-HCC patients. A more detailed description can be found in [5].

The goal of this study was to find features or feature-sets which were significantly differentially expressed between HCC group and control group. Hence, those features could be treated as potential diagnostic markers of the disease. Exploratory data analysis showed that some pairwise Pearson’s correlation coefficients were as high as 0.99. Hence, some features might correspond to the same metabolite with different ions (like H and Na adducts) in the MS-based metabolomics study. The m/z and retention time information in the dataset supported this assertion. Based on this observation, we applied the grouping algorithm using Pearson’s correlation described in the Methods Section to group those highly correlated features into the same feature-set. Other grouping schemes are also possible depending on the purpose of the study. The threshold correlation value for grouping was set to be 0.95. Because this dataset contained only 1388 features, there was not a huge need to use a low correlation threshold value to reduce the number of individual tests. Under this threshold value, all 1388 features were grouped as 1130 feature-sets. Among those 1130 feature-sets, 1064 of them contained a single feature and the biggest set contained 56 features. For each feature-set, a distance-based kernel score test and a stratified kernel score test were applied. For ease of presenting the result, we only applied the Wilcoxon signed-rank test. There are two major reasons for this. First, it is a robust test in the sense that it requires fewer assumptions, while the other three non-kernel methods (Qual, Quant, T) need the linearity assumption. The true relationship between hepatocellular carcinoma and metabolites abundance level is unknown and possibly nonlinear. Hence, this robustness is desired in our real data analysis. Second, the Wilcoxon signed-rank test showed a better performance than the three parametric methods in most of scenarios in the simulation studies.

**Figure 3:**
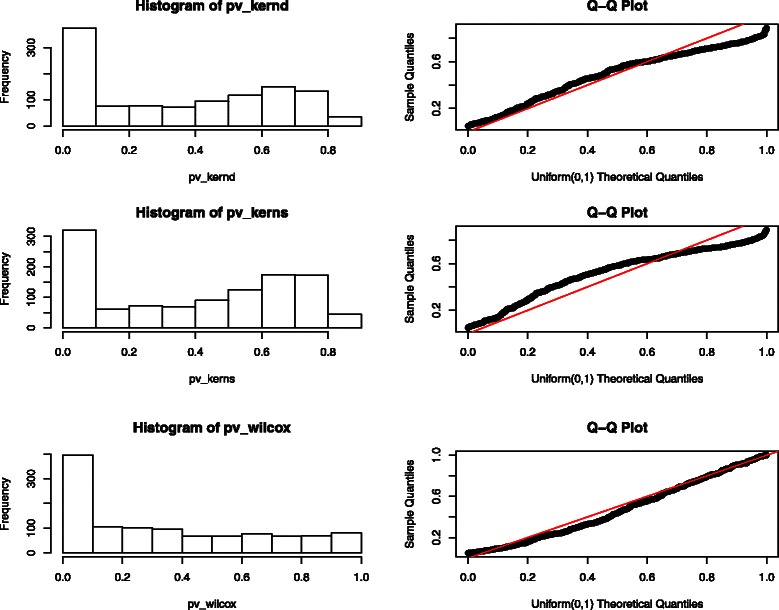
**P-values of different methods.** The left panel are the histograms of all p-values, and the right panel are the QQ-plot of those p-values greater than 0.05.

**Figure 4:**
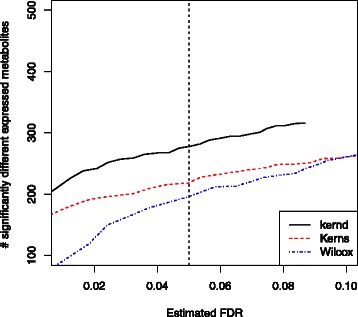
**Significance versus FDR.** Number of significantly differentially expressed metabolites versus FDR estimation on hepatocellular carcinoma data. The vertical dotted line has an estimated FDR of 0.05.

Figure 3 illustrates the p-values obtained from the three methods. We checked the distribution of those p-values greater than 0.05 using QQ-plot on the right panel in Figure 3. The deviations from the straight line were mostly minimal. Hence, those p-values greater than 0.05 were almost distributed as Uniform (0,1), and it was valid to use Eq. (9) to estimate FDR. Figure 4 shows curves of number of significantly differentially expressed metabolites versus FDR estimation. Each point in the curve corresponds to a cutoff value *c*. The y-axis is associated with the number of features with a p-value smaller than *c*, and the x-axis is the estimated $\hat {FDR}(c)$ using Eq. (9). The range of the cutoff value *c* was set to be (0,0.05) in Figure 4. Different *λ* values in Eq. (9) were used and those results were similar. The one presented in Figure 4 corresponds to *λ*=0.7. Based on Figure 4, a distance-based kernel score test had the best performance in that it could detect more significance at a given estimated FDR level than the other two methods. At an estimated FDR level lower than 0.1, our stratified kernel score test also outperformed the Wilcoxon signed-rank test. At an estimated FDR level of 0.05, 279, 218, 194 feature-sets were detected as significantly differentially expressed by distance-based kernel score test, stratified kernel score test and Wilcoxon test respectively. At a estimated FDR level of 0.01, the numbers of rejections from the three tests were 210, 163 and 86. Therefore, our kernel-based method had best performance in metabolomics differential expression analysis on this HCC data especially at a low FDR level. In this HCC dataset, 1064 out of 1130 feature-sets contain only one feature. There are a lot of tied zero values in those single-feature feature-sets. Those ties reduce the power of the Wilcoxon signed-rank test. Moreover, we also performed the grouping based on Spearman’s correlation. The results are shown in Section 2 in Additional file 1. The results obtained from grouping based on Spearman’s correlation are very similar with those using Pearson’s correlation. The same analysis we did here can also apply to the differential analysis using Spearman’s correlation-based groupings. Our kernel approach for differential expression analysis has a good performance irrespective of the grouping scheme.

Finally, [5] used a different error measure, the family-wise error rate (FWER), instead of FDR in their study. They used Bonferroni correction to control the FWER. That was, any individual test with a p-value smaller than *α*/*n* was rejected in order to achieve a FWER of *α*, where *n* was the number of individual tests. We were also interested in a comparison between their method and our kernel methods. In [5], about 30 significant features were determined by the Student t test with Bonferroni correction to control a family-wise error rate (FWER) of 0.05. We also applied Bonferroni correction at a FWER level of 0.05 to our methods, and found that 159, 125 and 34 feature-sets were significantly differently expressed by distance-based kernel score test, stratified kernel score test and Wilcoxon signed-rank test respectively. At a FWER of 0.05, the Wilcoxon test has similar performance with the Student t test in [5]. Our kernel-based approaches are much more powerful in that they can reject more hypotheses at the same level of statistical confidence. Further research of these significant features would provide new insights into the pathobiology of the disease.

## Conclusion

In this article, we have developed a kernel-based analysis for metabolomics data that is designed to test for differentially expressed metabolites. One challenge in metabolomics is the sparsity of the data matrix resulting from missing values. We extend the kernel-based score test widely used in genomics to metabolomics by introducing two new kernels. The new kernels can incorporate both the discrete nature as well as continuous nature of metabolomics data into differential expression analysis. Besides the advantage of being able to capture both aspects of information in metabolomics data, the p-value of kernel score test is almost continuous and can be fit into the existing FDR estimation framework proposed in [31]. In order to combine both presence/absense information as well as the quantitative information, some authors have applied two separate tests and then introduce some new approach to estimate a overall FDR measure [10]. Those methods are new and not well-studied compared with the one in [31]. In this sense, our kernel approach is more general and easily fits into the existing FDR framework.

We have also developed a data-driven algorithm to group metabolites into clusters as a preprocessing step to performing the association test. However, the criteria for selecting clusters based on correlation is *ad hoc*. Some other grouping schemes have also been proposed in the literature [27-29]. Further work is needed in order to provide more formal guidance on how to do metabolite grouping. The kernel-based approach has better performance relative to standard methods in both the simulated examples and the hepatocellular carcinoma dataset we examined. An implementation of the proposed kernel metabolomics differential analysis method in the R statistical computing environment is available at http://works.bepress.com/debashis_ghosh/60/.

## Additional file

Additional file 1 **Supplementary material.** The Supplementary material includes the proof of positive-definiteness of the distance-based kernel, and more simulation results on the HCC dataset.
