# Supplementary material for: Kernel approaches for differential expression analysis of mass spectrometry-based metabolomics data
Source: BMC Bioinformatics. 2015 Mar 11;16:77. doi: 10.1186/s12859-015-0506-3 (PMC4359587; doi:10.1186/s12859-015-0506-3)
Supplement: Supplementary file 1 — Supplementary material. The Supplementary material includes the proof of positive-definiteness of the distance-based kernel, and more simulation results on the HCC dataset. [file 12859_2015_506_MOESM1_ESM.pdf]

Supplementary material: Kernel approaches for differential expression analysis of mass spectrometry-based metabolomics data

Xiang Zhan*[1], Andrew D Patterson†[2], and Debashis Ghosh‡[3]

[1]*Department of Statistics, Pennsylvania State University*
[2]*Department of Molecular Toxicology, Pennsylvania State University*
[3]*Department of Biostatistics and Informatics, Colorado School of Public Health, University of Colorado Anschutz Medical Campus*

December 27, 2014

Abstract

This document is the Supplementary material for the submission titled "Kernel approaches for differential expression analysis of mass spectrometry-based metabolomics data" [2]. The supplementary material has two sections. The first one is about the proof of positive-definiteness of the distance-based kernel introduced in [2]. The second section contains some more simulation results on the liver cancer dataset HCC analyzed in [2]. Both the dataset and implementation of the proposed kernel method in the R statistical computing environment is available at `http://works.bepress.com/debashis_ghosh/60/`.

1 Distance-based kernel

We first show that function $d(x, y)$ defined in Eq. (1) is a well-defined distance metric.

$$d(x, y) = \sqrt{\sum_{i=1}^{p} I_{[\delta_{x_i} \neq \delta_{y_i}]} + \sum_{i=1}^{p} (x_i - y_i)^2}, \tag{1}$$

*`xyz5074@psu.edu`
†`adp117@psu.edu`
‡`debashis.ghosh@ucdenver.edu`; Corresponding author

where $x \equiv (x_1, \ldots, x_p)^T$, $y \equiv (y_1, \ldots, y_p)^T$ and $\delta_{x_i} = I_{[x_i \neq 0]}$. Based on the definition in (1), it is easy to see that following conditions hold:

- Non-negativity: $d(x, y) \geq 0$;
- Coincidence axiom: $d(x, y) = 0$ if and only if $x = y$;
- Symmetry: $d(x, y) = d(y, x)$.

In order to show that (1) is a well-defined distance metric, we only need to show that the triangle inequality also holds. Note that if $\delta_x \neq \delta_z$, then either $\delta_x \neq \delta_y$ or $\delta_y \neq \delta_z$. Hence the following indicator inequality holds:

$$I_{[\delta_x \neq \delta_z]} \leq I_{[\delta_x \neq \delta_y]} + I_{[\delta_y \neq \delta_z]}.$$

Then the triangle inequality holds in the following way:

$$\begin{aligned}
d(x,z)^2 &= \textstyle\sum_{i=1}^p I_{[\delta_{x_i} \neq \delta_{z_i}]} + \sum_{i=1}^p (x_i - z_i)^2 \\
&\leq \textstyle\sum_{i=1}^p I_{[\delta_{x_i} \neq \delta_{y_i}]} + \sum_{i=1}^p I_{[\delta_{y_i} \neq \delta_{z_i}]} \\
&+ \textstyle\sum_{i=1}^p (x_i - y_i + y_i - z_i)^2 \ (indicator\ inequality) \\
&= d(x,y)^2 + d(y,z)^2 + 2\textstyle\sum_{i=1}^p (x_i - y_i)(y_i - z_i) \\
&\leq d(x,y)^2 + d(y,z)^2 + 2\sqrt{\textstyle\sum_{i=1}^p (x_i - y_i)^2} \\
&\times \sqrt{\textstyle\sum_{i=1}^p (y_i - z_i)^2} \ (Cauchy - Schwarz\ inequality) \\
&\leq d(x,y)^2 + d(y,z)^2 + 2d(x,y)d(y,z) \\
&= [d(x,y) + d(y,z)]^2
\end{aligned}$$

which establishes the triangle inequality. Hence distance Eq.(1) is a well-defined distance metric. Now, we need to show that distance-based kernel

$$k_d(x, y) = \exp\{-\frac{d^2(x, y)}{\rho}\}, \rho > 0, \qquad (2)$$

is a well-defined kernel. That is, we need to show that for any n and a collection of n distinct points $x_1, \ldots, x_n \in \mathcal{X}$ (the input space), the kernel matrix K with $K_{ij} = k_d(x_i, x_j)$ is positive definite. The following definition and lemmas are helpful.

Definition 1 (Negative Definite Function). Let $\mathcal{X}$ be a nonempty set. A function $d : \mathcal{X} \times \mathcal{X} \to R$ is called a *negative definite function* if d is symmetric $d(x, y) = d(y, x)$ and

$$\sum_{i=1}^n \sum_{j=1}^n a_i a_j d(x_i, x_j) \leq 0,$$

for any $x_1, \ldots, x_n (n \geq 2) \in \mathcal{X}$ and $a_1, \ldots, a_n \in R$ with $\sum a_i = 0$.
Note: Based on this definition, it is easy to see that, if k is positive

definite, then $d = -k$ is negative definite.

Lemma 1. Let $d(x, y)$ be a symmetric function on $\mathcal{X}$. Fix $x_0 \in \mathcal{X}$ and define

$$k(x, y) = -d(x, y) + d(x, x_0) + d(x_0, y) - d(x_0, x_0).$$

Then d is negative definite if and only if k is positive definite.
PF: "If" part: Consider an arbitrary $a_1, \dots, a_n \in R$ with $\sum a_i = 0$. Because k is positive definite,

$$\begin{aligned} 0 &\leq \textstyle\sum_{i,j} a_i a_j k(x_i, x_j) \\ &= \textstyle\sum_{i,j} a_i a_j [-d(x_i, x_j) + d(x_i, x_0) + d(x_0, x_j) - d(x_0, x_0)] \\ &= -\textstyle\sum_{i,j} a_i a_j d(x_i, x_j) \end{aligned}$$

That is,

$$\sum_{i=1}^{n} \sum_{j=1}^{n} a_i a_j d(x_i, x_j) \leq 0,$$

which shows that d is negative definite.

"Only if" part: Consider arbitrary $(x_1, \dots, x_n) \in \mathcal{X}^n$ and $(a_1, \dots, a_n) \in R^n$. Define $a_0 = -\sum_{i=1}^{n} a_i$. By the negative definiteness of d,

$$0 \geq \sum_{i=0}^{n} \sum_{j=0}^{n} a_i a_j d(x_i, x_j) = -\sum_{i=1}^{n} \sum_{j=1}^{n} a_i a_j k(x_i, x_j).$$

That is,

$$\sum_{i=1}^{n} \sum_{j=1}^{n} a_i a_j k(x_i, x_j) \geq 0,$$

which established the positive definiteness of k. Q.E.D.

Lemma 2. (1) If $k_i, i = 1, 2, \dots$ are positive definite kernels, then the following are positive definite kernels:

- Positive combination: $ak_1 + bk_2$ where $a, b \geq 0$;
- Product: $k_1 k_2$;
- Limit: $\lim_{i \to \infty} k_i$, assuming the limit exists.

(2) If $k_i, i = 1, 2, \dots$ are negative definite kernels, then the following are negative definite kernels:

- Positive combination: $ak_1 + bk_2$ where $a, b \geq 0$;

- Limit: $\lim_{i\to\infty} k_i$, assuming the limit exists.

Note: The proof of this lemma can be found in [1]. An application of this Lemma is that: If $k(x,y)$ is positive definite, by this lemma and Taylor expansion, $e^{k(x,y)}$ is also positive definite.

Lemma 3. Let $\mathcal{X}$ be a nonempty set, and $d : \mathcal{X} \times \mathcal{X} \to R$ be a kernel. d is negative definite if and only if $k(x,y) = exp(-\rho d(x,y))$ is positive definite for all $\rho > 0$.
PF: "If" part:

$$d(x,y) = \lim_{\rho \downarrow 0} \frac{1 - exp(-\rho d(x,y))}{\rho}$$

Since $exp(-\rho d(x,y))$ is positive definite, $-exp(-\rho d(x,y))$ is negative definite. It is easy to see that constant 1 is negative definite. Therefore by Lemma 2, we can conclude that $d(x,y)$ is negative definite.

"Only if" part: By Lemma 2, if d is negative definite and $\rho > 0$, then ρd is negative definite. So only need to show the conclusion holds for $\rho = 1$. That is to show that $\exp(-d(x,y))$ is positive definite. Take $x_0 \in \mathcal{X}$ and define

$$k_1(x,y) = -d(x,y) + d(x,x_0) + d(x_0,y) - d(x_0,x_0)$$

By Lemma 1, $k_1(x,y)$ is positive definite. Taking exponential on both sides, we have

$$\begin{aligned} \exp(-d(x,y)) &= \exp(d(x_0,x_0)) \exp(k_1(x,y)) \\ &\times \exp(-[d(x,x_0) + d(x_0,y)]) \end{aligned}$$

Note that $exp(d(x_0,x_0))$ is a positive constant, $exp(k_1(x,y))$ is positive definite, and $exp(-[d(x,x_0)+d(x_0,y)]) = exp(-d(x,x_0))exp(-d(y,x_0))$ can be shown to be positive definite by definition. Hence by Lemma 2, the product $exp(-d(x,y))$ of three positive definite functions is also positive definite. Q.E.D.

Theorem The distance-based kernel defined in Eq. (2) is positive definite.
PF: Consider arbitrary $x_1, \ldots, x_n \in \mathcal{X}$ and coefficients $(a_1, \ldots, a_n)$ with $\sum a_i = 0$.

$$\sum_{i=1}^{n}\sum_{j=1}^{n} a_i a_j d^2(x_i,x_j) = \sum_{i=1}^{n}\sum_{j=1}^{n} a_i a_j [(\delta_{x_i} - \delta_{x_j})^2 + (x_i - x_j)^2] \equiv Q_1 + Q_2,$$

where

$$Q_1 = \sum_{i=1}^{n}\sum_{j=1}^{n} a_i a_j[(\delta_{x_i})^2 + (\delta_{x_j})^2 - 2\delta_{x_i}\delta_{x_j}] = -2(\sum_{i=1}^{n} a_i\delta_{x_i})^2 \leq 0$$

Similarly,

$$Q_2 = \sum_{i=1}^{n}\sum_{j=1}^{n} a_i a_j[(x_i)^2 + (x_j)^2 - 2x_i x_j] = -2(\sum_{i=1}^{n} a_i x_i)^2 \leq 0$$

That is,

$$\sum_{i=1}^{n}\sum_{j=1}^{n} a_i a_j d^2(x_i, x_j) \leq 0,$$

Therefore function $d^2(x,y)$ is negative definite. Therefore by Lemma 3, $k_d(x,y) = exp(-\rho d^2(x,y))$ is positive definite for any $\rho > 0$. Hence we establish the positive definiteness of the distance-based kernel.

2 More results on HCC data

We also performed groupings on the real HCC dataset [2] using Spearman's correlation. The grouping algorithm is described in the Method Section in [2]. A total of 1388 features were grouped as 1339 feature-sets using the threshold of 0.95. Among those 1339 feature-sets, 1317 of them contained a single feature and the largest set contained 11 features. We applied differential analysis on each feature-sets. Figure 1 illustrates the p-values obtained from the distance-based kernel score test (kernd), stratified kernel score test (kerns) and Wilcoxon signed-rank test (wilcox) respectively. As shown in Figure 1, the estimated null p-values (p-values greater than 0.05) were almost distributed as Uniform (0,1). Hence, we used the method in [3] to estimate FDR. Figure 2 shows curves of number of significantly differentially expressed metabolites versus FDR estimation. Each point in the curve corresponds to a cutoff value c. The y-axis is associated with the number of features with a p-value smaller than c, and the x-axis is the estimated $F\hat{D}R(c)$ using [3]. The range of the cutoff value c was set to be $(0, 0.05)$ in Figure 2. Different λ values in Eq. (9) in [2] were used and those results were similar. The one presented in Figure 2 corresponds to $\lambda = 0.7$. A similar result as in [2] is also observed in Figure 2. And the same analysis in the Real data section in [2] also applies here.

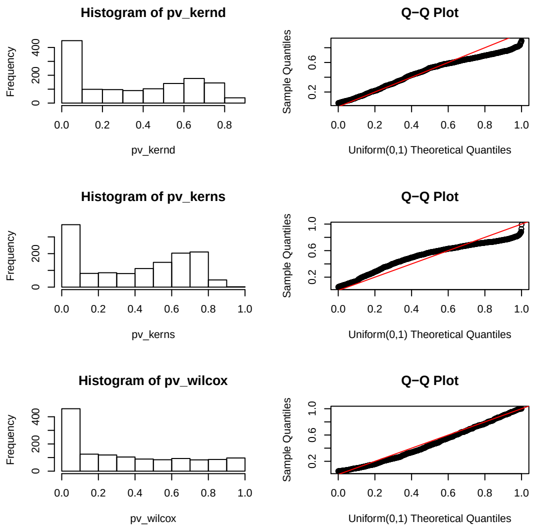

Figure 1: P-values of different methods.The left panel are the histograms of all p-values, and the right panel are the QQ-plot of those p-values greater than 0.05.

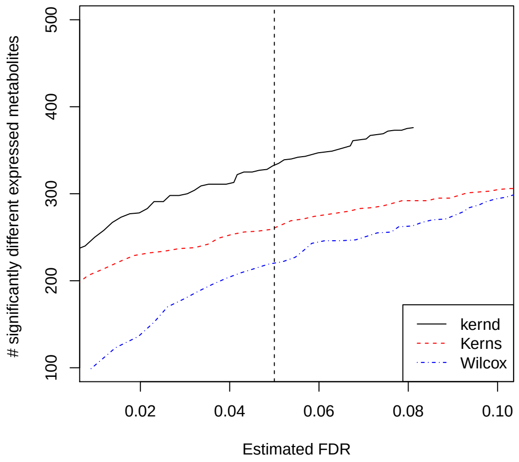

Figure 2: Significance versus FDR. Number of significantly differentially expressed metabolites versus FDR estimation on hepatocellular carcinoma (HCC) data. The Spearman's correlation is applied when grouping features in HCC data. The vertical dotted line has an estimated FDR of 0.05.

References

[1] Berg, C., Christensen, J. P. R., and Ressel, P. (1984) *Harmonic Analysis on Semigroups.* New York: Springer.

[2] Zhan, X., Patterson, A. D., and Ghosh, D. (2015) *Kernel approaches for differential expression analysis of mass spectrometry-based metabolomics data.* BMC Bioinformatics, to appear.

[3] Storey, J. D., and Tibshirani, R. (2003). *Statistical significance for genomewide studies.* Proceedings of the National Academy of Sciences, 100(16), 9440-9445.
